# Type II Endometrial Cancer Overexpresses NILCO: A Preliminary Evaluation

**DOI:** 10.1155/2017/8248175

**Published:** 2017-06-04

**Authors:** Danielle Daley-Brown, Gabriela Oprea-Iles, Kiara T. Vann, Viola Lanier, Regina Lee, Pierre V. Candelaria, Alexander Quarshie, Roland Pattillo, Ruben Rene Gonzalez-Perez

**Affiliations:** ^1^Department of Microbiology, Biochemistry and Immunology, Morehouse School of Medicine, Atlanta, GA, USA; ^2^Department of Pathology and Laboratory Medicine, Emory University, Atlanta, GA, USA; ^3^Department of Obstetrics and Gynecology, Morehouse School of Medicine, Atlanta, GA, USA; ^4^Department of Community Health & Preventive Medicine, Morehouse School of Medicine, Atlanta, GA, USA

## Abstract

**Objective:**

The expression of NILCO molecules (Notch, IL-1, and leptin crosstalk outcome) and the association with obesity were investigated in types I and II endometrial cancer (EmCa). Additionally, the involvement of NILCO in leptin-induced invasiveness of EmCa cells was investigated.

**Methods:**

The expression of NILCO mRNAs and proteins were analyzed in EmCa from African-American (*n* = 29) and Chinese patients (tissue array, *n* = 120 cases). The role of NILCO in leptin-induced invasion of Ishikawa and An3ca EmCa cells was investigated using Notch, IL-1, and leptin signaling inhibitors.

**Results:**

NILCO molecules were expressed higher in type II EmCa, regardless of ethnic background or obesity status of patients. NILCO proteins were mainly localized in the cellular membrane and cytoplasm of type II EmCa. Additionally, EmCa from obese African-American patients showed higher levels of NILCO molecules than EmCa from lean patients. Notably, leptin-induced EmCa cell invasion was abrogated by NILCO inhibitors.

**Conclusion:**

Type II EmCa expressed higher NILCO molecules, which may suggest it is involved in the progression of the more aggressive EmCa phenotype. Obesity was associated with higher expression of NILCO molecules in EmCa. Leptin-induced cell invasion was dependent on NILCO. Hence, NILCO might be involved in tumor progression and could represent a new target/biomarker for type II EmCa.

## 1. Background

Endometrial cancer (EmCa) is the most common gynecological malignancy of the female reproductive tract [1]. As of 2015, there were 54,870 new EmCa cases reported and 10,170 deaths reported in the United States [2]. The incidence of EmCa is higher in well-developed countries and countries with high obesity rates [3]. Caucasian patients are at a higher risk of developing endometrial cancer when compared to African-American patients in the US. In 2014, EmCa incidence rate of Caucasian patients was 24.8 per 100,000 women, whereas in African-American women it was 20.9 per 100,000 women [3]. There are still controversial opinions on the categorical classification of types I and II EmCa. However, type I EmCa is estrogen dependent. In contrast, type II EmCa is estrogen independent, more aggressive, shows poor prognosis, and is usually associated with endometrial atrophy [1, 4].

Obesity, characterized as having a body mass index (BMI) of thirty or higher, is a major risk factor for EmCa and is a comorbid condition found in approximately 17–46% of all EmCa cases [5]. The heaviest women are at the highest risk of EmCa [6]. Studies have shown that overweight women were twice as likely to develop the disease compared to normal-weight women, while obese women are four times more likely to develop EmCa [7]. Interestingly, African-American women show the highest incidence of obesity and type II EmCa and are more likely to die from this disease. However, whether obesity is a driver for EmCa is not very well understood [1, 8]. The reason(s) for this cancer health disparity is unknown [8]. Obese individuals have high serum levels of leptin, an adipokine strongly linked to poor prognosis and higher incidence of several cancer types, including breast, colon, pancreas, stomach, and thyroid cancer among others [9]. High circulating levels of leptin in obese people correlate to the size of adipose tissue and generally to BMI [10]. The main function of leptin is the regulation of appetite and energy balance. Leptin exerts its effects on energy balance through specific signaling pathways in hypothalamic neurons that express the leptin receptor, OB-R [10]. However, obesity is characterized by hypothalamic unresponsiveness to leptin signals, which is known as leptin resistance [10].

Leptin regulates glucose homeostasis, growth, reproduction, and immune response [11]. Leptin's pleiotropic functions also involve angiogenic, inflammatory, and antiapoptotic effects, which are important for cells overexpressing OB-R, including cancer cells [12]. Moreover, several reports have shown a link between leptin signaling and the development of cancer stem cells and resistance to chemotherapeutics. Therefore, leptin is a growth, angiogenic, and survival factor for several types of tumors [12].

Leptin is secreted by adipocytes as well as cancer cells [13]. Therefore, leptin produced by adipocytes and cancer cells could act in an autocrine and paracrine manner to promote proliferation, migration, survival, invasion and proinflammatory processes in tumor cells, and tumor angiogenesis [14]. Accumulating evidence strongly suggest that high levels of leptin and OB-R found in tumor tissues are associated with metastasis and decreased survival rates of breast cancer patients [10, 14, 15]. OB-R has several isoforms. OB-Rl or OB-Rb is the long isoform with full signaling capabilities. OB-Ra, the short form of the receptor, has limited signaling capabilities and has been found in EmCa cells [10, 16].

Notch signaling is a hallmark of several cancers. Aberrant activation of the Notch signaling pathway can be initiated through the abnormal expression of Notch ligands, receptors, and target genes; all of which have been reported in several solid tumors, including breast, prostate, and pancreatic tumors. However, the involvement of specific Notch receptors in cancer is dependent on cell type [17]. Similarly, IL-1 is an inflammatory and proangiogenic cytokine that can promote tumor angiogenesis, growth, and metastasis [18]. IL-1 and leptin induce VEGF in cancer [19]. In turn, VEGF can regulate the expression of Notch signaling components [20]. Providing a feedback mechanism, Notch signaling in turn can alter expression levels of all three VEGF receptors [14]. Leptin also induces VEGFR in cancer cells [21] and promotes angiogenic features in endothelial cells via upregulation/transactivation of VEGFR and downstream expression/activation of Notch. Thus, high levels of leptin found in overweight and obese patients might lead to increased angiogenesis by activating VEGFR-Notch signaling crosstalk in endothelial cells [22].

It was previously reported that leptin signaling induced a complex molecular crosstalk in breast cancer that involved Notch and IL-1 signaling (NILCO). Interestingly, the blockade of the IL-1 system, in breast cancer, inhibited leptin's effects on Notch expression, suggesting that leptin-induced Notch activation is reliant on IL-1 signaling [14]. NILCO is a driver of breast cancer cell proliferation, migration, and survival. Moreover, NILCO induces essential proangiogenic molecules (i.e., VEGF/VEGFR-2) and Notch targeted genes (i.e., Survivin and Hey2, and so forth) [14, 15, 23]. Data generated from obese mice hosting syngeneic mammary tumors showed leptin signaling induces Notch in vivo, which was linked to tumor growth and angiogenesis. These studies further suggest that obesity accelerates breast cancer progression via leptin induction of NILCO [15].

Likewise, EmCa cells express IL-1, leptin/OB-R, and Notch [24–26]. In addition, leptin increased the levels of VEGF in EmCa. However, it is unknown whether the expression of NILCO components is associated with EmCa progression, aggressiveness, and prognosis. The main objectives of the current study were to determine whether NILCO components are differentially expressed in the more aggressive type II EmCa and whether obesity may influence this outcome. Current data suggest that NILCO components are expressed higher in type II EmCa, and their level of expression correlates to obesity status. These data suggest that NILCO may be involved in EmCa progression and prognosis. Moreover, current findings suggest that NILCO might be used as a novel biomarker for type II EmCa.

## 2. Methods

### 2.1. Cell Lines, Reagents, and Antibodies

An3ca EmCa cells and Penicillin-Streptomycin mixture were purchased from American Type Culture Medium (ATCC, Manassas, VA). Ishikawa EmCa cells, N-[N-(3, 5-difluorophenacetyl)-L-alanyl]-S-phenylglycine t-butyl ester (DAPT), and dimethyl sulfoxide (DMSO), monoclonal antibody for Notch1 (N6786) and monoclonal antibody anti-GAPDH (G8795), were purchased from Sigma-Aldrich (St. Luis, MO). Dulbecco's Modified Eagle's medium (DMEM) and fetal bovine serum (FBS) were from Gemini Bio Products (West Sacramento, CA). Polyclonal antibodies for Notch4 (sc-5594), JAG1 (sc-8303), IL-1R tI (sc-688), and OB-R (sc-8325); long and short isoforms and western blot positive controls for Notch1 (F9 cell lysates), Notch4 (JAR cell lysate), JAG1 (raw cell lysates), OB-R (COLO 320DM), and IL-1R tI (CCRF) were obtained from Santa Cruz Biotechnology, Inc. (Dallas, TX). Polyclonal DLL4 (ab7280), Notch2 (ab72803), and Notch3 (ab23426) antibodies were purchased from Abcam Inc. (Cambridge, MA). Monoclonal Survivin (71G4B7) antibody was obtained from Cell Signaling Technology (Danvers, MA). Polyclonal antibody for Hey2 (AB5716) was obtained from Millipore (Billerica, MA). Hematoxylin was purchased from Dako Corporation (Carpinteria, CA). Human recombinant leptin and leptin and IL-1*β* ELISA kits were purchased from R&D Systems (Minneapolis, MN). The leptin peptide receptor antagonist 2 (LPrA2) was synthesized and purified as previously described [21]. LPrA2 was bound to iron oxide nanoparticles (IONP) to increase its effectiveness (Ocean Nanotech, San Diego, CA) [27]. All other reagents were obtained from Sigma.

### 2.2. Patients and Specimens

The endometrial samples were obtained from Grady Memorial Hospital, Atlanta, GA, under IRB approved protocols (Grady Memorial Hospital and Morehouse School of Medicine, Atlanta, GA) and a patient's written informed consent. Nonmalignant and EmCa samples were obtained from African-American women (*n* = 29; 17 obese and 12 lean) undergoing uterine surgery. Tumor samples were staged and graded by a pathologist and consist of type I EmCa (*n* = 12; 7 obese and 5 lean), all endometrioid adenocarcinomas; and type II EmCa (*n* = 17; 10 obese and 7 lean) were papillary adenocarcinoma (*n* = 4), papillary serous adenocarcinoma (*n* = 8), carcinosarcoma (*n* = 1), and mixed clear cell and serous adenocarcinoma (*n* = 4). Each EmCa sample had a paired control sample from adjacent nontumor endometrial tissue, as determined by pathologists. Additionally, EmCa tissue arrays from Chinese patients (EmCa, *n* = 120, and nonmalignant endometria, *n* = 30) were obtained from US Biomax, Inc. (Rockville, MD). Tissue cores were fixed in formalin. EmCa features from biopsies obtained from Chinese patients included age, TNM, and tumor grading. However, no body weight or body mass index (BMI) information was available for Chinese patients. It was also unknown if some nonmalignant samples were obtained from adjacent normal endometria from the Chinese EmCa patients. Each endometrial tissue array from Chinese donors contained type I EmCa (*n* = 97) and type II EmCa (*n* = 23) and nonmalignant endometrial tissues: normal (*n* = 23) and hyperplasia (*n* = 7) samples that were classified by a pathologist. A total of 120 cores were scored using the HSCORE method described below.

### 2.3. Immunohistochemistry (IHC)

Paraffin slides of EmCa and nonmalignant tissues (5 *μ*m) were used for IHC. Specific antibodies were used to analyze the following antigens: Notch1, Notch2, Notch3, Notch4, JAG1, DLL4, IL-1R tI, OB-R, Survivin, and Hey2. Tissue sections were deparaffinized, and sodium citrate buffer (pH6, 10mM) was used for antigen retrieval at 95°C for 30 minutes. Primary antibodies were diluted in PBS as follows: Notch1 (1 : 50), Notch2 (1 : 50), Notch3 (1 : 50), Notch4 (1 : 50), JAG1 (1 : 50), DLL4 (1 : 50), IL-1R tI (1 : 50), OB-R (1 : 50), Survivin (1 : 400), and Hey2 (1 : 500), and tissue sections were incubated overnight at 4°C. Slides were incubated with secondary biotinylated antibody using the Immunocruz ABC staining system (Santa Cruz Biotech) for 45 minutes at room temperature. The slides were counterstained with hematoxylin and mounted with Vecta Mount Mounting Medium (Vecta). Antigen staining intensity was evaluated using the HSCORE method. Each slide was evaluated by two independent observers, who counted 100 cells in three different optical fields. HSCORE was calculated using the equation ∑pi (*i* + 1), where “*i*” is the intensity with a value of 0, 1, 2, or 3 (negative, weak, moderate, or strong, resp., and “pi” is the percentage of stained cells for each intensity) [23]. Negative control slides (no primary antibody) were incubated only with secondary antibodies (anti-rabbit and anti-mouse, Santa Cruz).

### 2.4. Western Blot

Frozen tissue sections from African-American patients were used for Western blot (WB) analyses. All available frozen sections for western blot (*n* = 8, type I and *n* = 6, type II) were pooled to eliminate bias during the immunoblot analyses. All samples used in WB were represented in the original IHC cohort. Fifty mg of EmCa and nonmalignant tissues were lysed and extracted using radioimmunoprecipitation assay (RIPA) buffer containing a protease/phosphatase inhibitor cocktail (Sigma). The Bradford Assay was used to determine the protein concentrations of tissue lysates. Fifty *μ*g of protein was used for WB. The same antibodies used in immunohistochemistry analysis were used for WB, and a 1 : 200 dilution of primary antibody was used. Specific positive controls (Santa Cruz Biotech) for each primary antibody were used for more accurate identification of specific antigens. WB results were normalized using *β*-actin as loading control. The NIH Image program (Image J) was used for quantitative analysis of protein bands. Band density values from type II EmCa were calculated assuming 100% of expression in type I EmCa. Representative data were derived from biological triplicates (mean+standard error).

### 2.5. ELISA

Type I and type II EmCa tissues from African-American patients were mechanically disrupted with RIPA buffer to produce tumors lysates. Human ELISA kits (R&D Systems) were used to determine leptin and IL-1*β* concentrations in tumor lysates per the manufacturer's instructions. RIPA buffer was used as control. ELISA sensitivities were 7.8pg/mL for leptin and 1pg/mL for IL-1*β*.

### 2.6. Real-Time PCR

EmCa biopsies from African-American patients (*n* = 8, type I and *n* = 6, type II) used for WB were analyzed by real-time PCR analyses (qPCR) of NILCO components. RNA was extracted and purified from 50 mg of frozen EmCa tissues to obtain cDNA for qPCR as described elsewhere [15]. The following primers were obtained from (Invitrogen, Carlsbad, CA): Notch1 forward: 5′-cactgtgggcgggtcc-3'and reverse: 5′-gttgtattggttcggcaccat-3′; Notch2 forward: 5′-aatccctgactccagaacg-3′ and reverse: 5′-tggtagaccaagtctgtgatg-3′; Notch3 forward: 5′-tgaccgtactggcgagact-3′ and reverse: ccgcttggctgcatcag-3′; Notch4 forward: 5′-tagggctccccagctctc3'and reverse: 5′-ggcaggtgcccccatt-3′; JAG1 forward: 5′-gactcatcagccgtgtctca-3′ and reverse: 5′-tggggaacactcacactcaa-3′; DLL4 forward: tgctgctggtggcacttt-3′ and reverse: 5′-cttgtgaggtgcctggtt-3′; IL-1R tI forward: 5′-gccaagagttctttaggtgcc-3'and reverse: 5′-ctcactgcaacctccgtctc-3′; OB-R forward: 5′-gctattttgggaagatgt-3′ and reverse: 5′-tgcctgggcctctatctc-3′; Survivin forward: 5′-gcccagtgtttcttctgctt3' and reverse: 5′-cctcccaaagtgctggtatt-3′; Hey2 forward: 5′-aaaaagctgaaatattgcaaat-3′ and reverse: 5′-gtaccgcgcaacttctgtt-3′; and GAPDH forward: 5′-agggctgcttttaactctggt-3'and reverse: 5′-ccccacttgattttggaggga-3′. qPCR conditions and relative expression values (R) were calculated as described previously [15] Representative data were derived from biological triplicates (mean + standard error).

### 2.7. Cell Invasion Assay

EmCa cells were cultured in Dulbecco's Modified Eagle's medium (DMEM), 10% FBS and 1% Penicillin-streptomycin. Ishikawa (derived from type I EmCa) and An3ca (derived from type II EmCa) cells (5 × 10^4^) [28] suspended in starvation medium (no FBS) were added to the upper chamber of an insert coated with matrigel (6.4 mm diameter, 8 mm pore size; Corning™ BioCoat™ Matrigel™ Invasion Chamber; BD Biosciences, San Jose, CA). *Inhibition of NILCO* were as follows: (1) *γ-secretase inhibition*: the upper chamber contained cells in basal medium plus 0.1% DMSO (basal) or DAPT (20 *μ*M DAPT/0.1% DMSO); (2) *IL-1 inhibition*: the upper chamber contained cells in basal medium with 1 *μ*g/ml of anti-IL-1R tI antibody; and (3) *leptin inhibition*: the upper chamber contained cells in basal medium with leptin receptor antagonist (0.0036 pM IONP-LPrA2). The inserts were placed in a 24-well plate containing starvation medium with or without 1.2nM leptin. Invasion assays were carried out for 24 h. Then, the cells in the lower side of the insert were fixed with 3.7% formaldehyde. Cells were stained with hematoxylin, and cells on the upper side of the insert were removed with a cotton swab. Six randomly selected fields (×10 objective) were photographed, and the migrated cells were counted [14].

### 2.8. Statistical Analysis

Statistical significance was established with the STATA statistical software package using ANOVA, two-tailed Student's *t*-test, and a Chi-square test or Fisher's exact test. *p* values were 2-sided, and a *p* value of less than 0.05 was considered statistically significant.

## 3. Results

### 3.1. EmCa Tissue Biopsies and Tissue Arrays

Table 1 summarizes the clinicopathological and histological characteristics of EmCa from African-American and Chinese patients. The cohort of African-American women was recruited from those patients currently attending to the Gynecological Medical services in the Grady Memorial Hospital, but not selection criteria was applied. It is unknown how the cohort of Chinese patients was selected as endometrial biopsies that were supplied by a commercial source (US Biomax).

Endometrial biopsies from African-American patients showing histopathological criteria of type I EmCa (*n* = 12) and type II EmCa (*n* = 17) were further classified using FIGO system and grading [29, 30]. Grades and stages showed significant differences between type I and type II EmCa (*p* = 0.0010 and 0.0051, resp.). As expected, type I EmCa was low grade (grades 1 and 2), while type II were high grade (grade 3). Likewise, roughly 75% of type I EmCa were low stage (stage I), but 65% of type II were high stage (stages III and IV). African-American patients with type I and type II EmCa showed significant differences between age (*p* = 0.0076), with type II EmCa patients showing a higher mean age. Noticeably, the mean BMI between patients having type I or type II EmCa were significantly different, with type II EmCa showing higher BMI (type I: 29.81 and type II: 34.17).

The histopathological characteristics of EmCa from Chinese patients are also shown in Table 1. All endometrial biopsies were evaluated and classified as type I (*n* = 97) and type II (*n* = 23) EmCa. Similarly, as the case of EmCa from African-American patients, type I and type II EmCa from Chinese patients showed significant differences in tumor grade and stage (*p* < 0.001). Most type I EmCa from Chinese patients were grade 1 (45%), whereas type II EmCa were mainly grade 3 (52%). In contrast to the EmCa African-American patients, mean ages of Chinese patients were not significantly different when compared to type I and type II EmCa.

### 3.2. Detection of NILCO and Targets in EmCa Tissues

**Figure 1:**
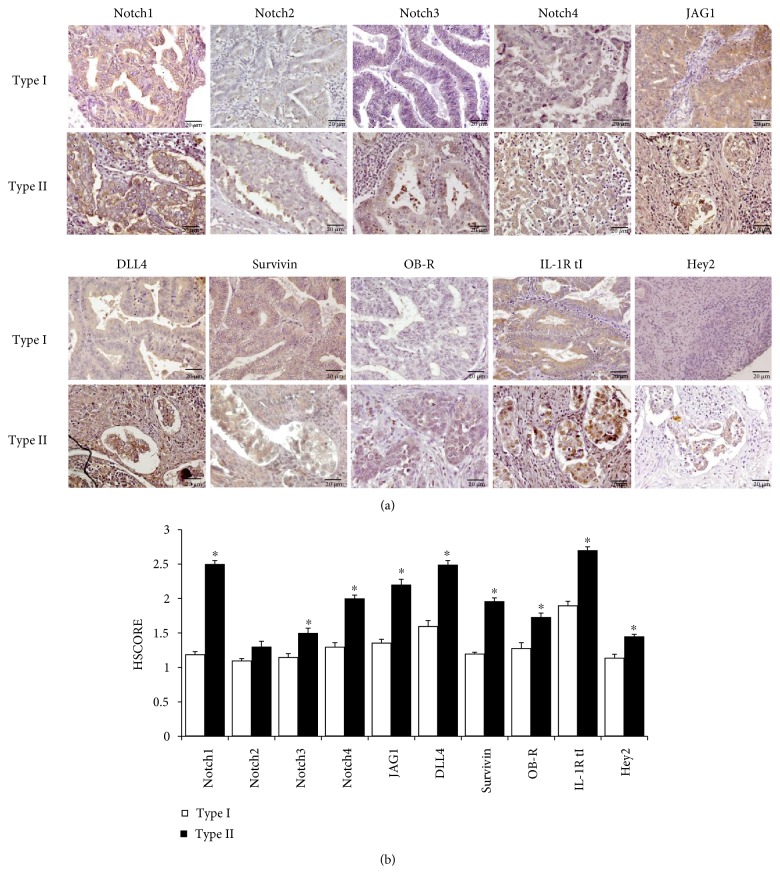
Immunohistochemical (IHC) detection of NILCO and targets in EmCa from African-American women. (a) Representative IHC pictures from type I (*n* = 7; 41%) and type II (*n* = 10; 59%) EmCa from obese African-American women. (b) Histogram of the semiquantitative HSCORE values. Brown color characterizes positive staining. Magnification ×40. ^∗^*p* < 0.05.

Figure 1(a) shows representative images of IHC staining of NILCO antigens examined in type I and type II EmCa from obese African-American patients. There were evident differences in the expression of Notch receptors, ligands, and targets between type I and type II EmCa. Noticeably, higher expression of Notch receptors, ligands, and targets were found in the type II, especially Notch1, Notch4, DLL4, and IL-1R tI.

**Figure 2:**
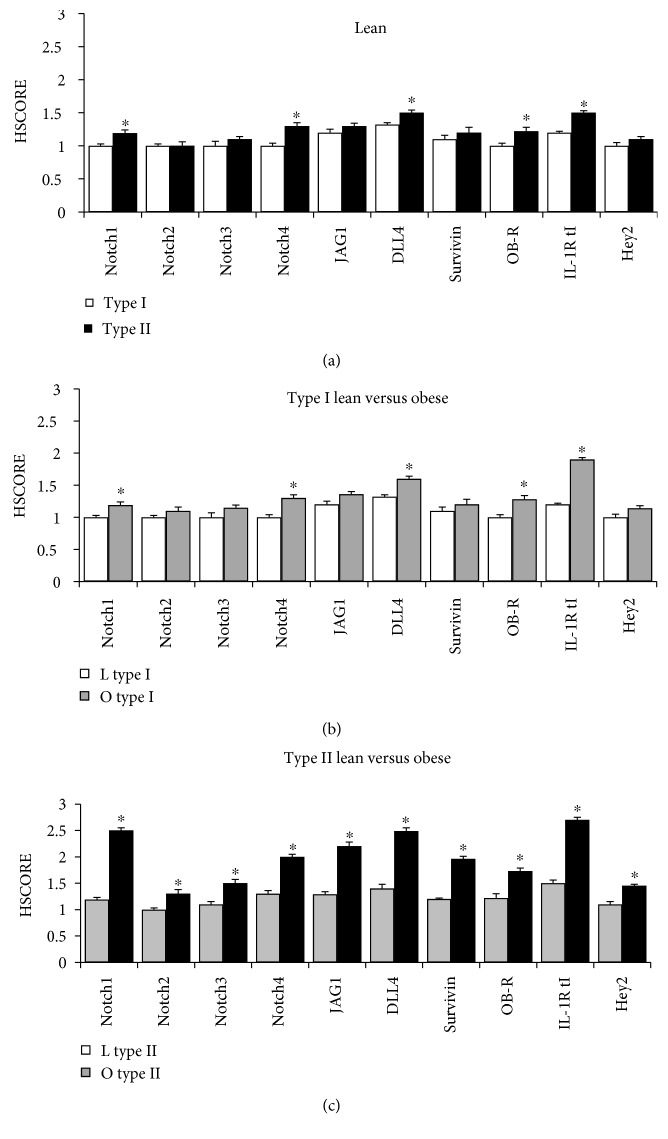
HSCORE values from lean and obese African-American women. (a) HSCORE of lean type I (*n* = 5; 42%) versus lean type II (*n* = 7; 58%). (b) HSCORE of lean type I (*n* = 5; 42%) versus obese type I (*n* = 7; 41%). (c) HSCORE of lean type II (*n* = 7; 58%) versus obese type II (*n* = 10; 59%). The expression levels of NILCO components and targets were analyzed in EmCa from lean (*n* = 12) versus obese (*n* = 17) women. ^∗^*p* < 0.05.

A representative histogram of the HSCOREs obtained from semiquantitative analyses of IHC staining of obese type I (*n* = 7, 41%) and type II (*n* = 10, 59%) EmCa from African-American patients is shown in Figure (1b). Interestingly, Notch1, Notch4, JAG1, DLL4, Survivin, OB-R, and IL-1R tI showed remarkably higher expression in type II EmCa (*p* = 0.001). A similar trend was observed in the lean patients (Figure 2(a)) with Notch4, OB-R, and IL-1R tI showing the greatest expression in type II EmCa (*n* = 7, 58%) (*p* < 0.05). When comparing lean and obese in the type I tumor only (Figure 2(b)), significantly higher expression in Notch1, Notch4, DLL4, OB-R, and IL-1R tI were found in obese patients (*p* < 0.05). Similarly, all NILCO components showed significantly higher expression in obese type II patients, especially in Notch1, Notch4, JAG1, DLL4, Survivin, and IL-1R tI (*p* < 0.05) (Figure 2(c)).

**Figure 3:**
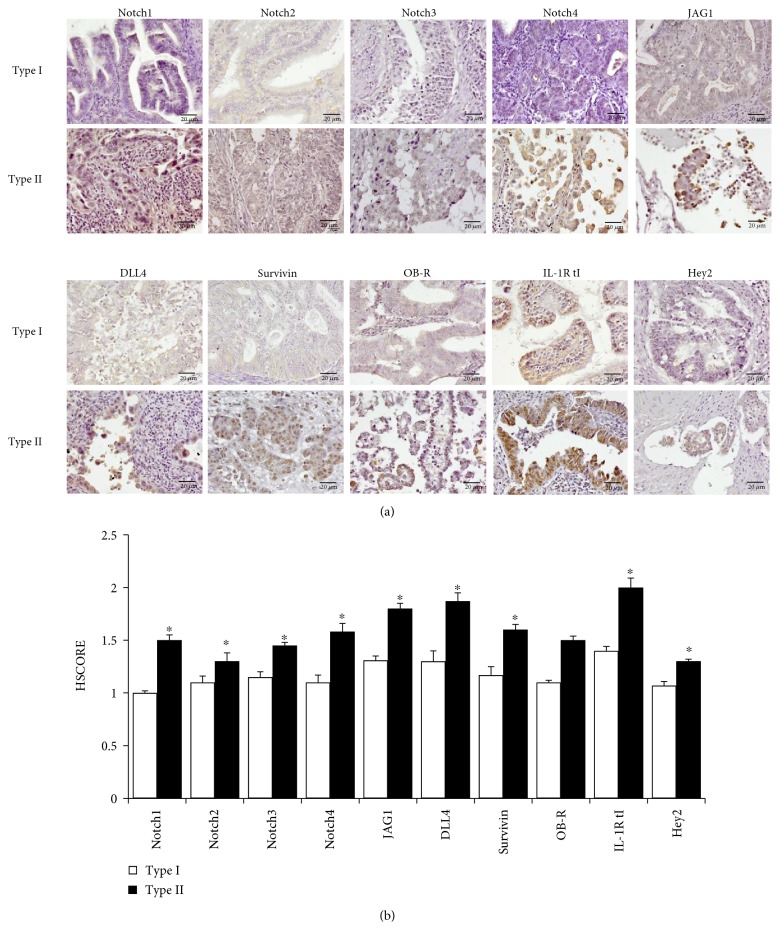
Immunohistochemical (IHC) detection of NILCO and targets in EmCa tissue microarrays from Chinese women. (a) Representative IHC pictures from type I (*n* = 97; 81%) and type II (*n* = 23; 19%) EmCa from Chinese women. (b) Histogram of the semiquantitative HSCORE values. Brown color characterizes positive staining. Magnification ×40. ^∗^*p* < 0.05.

Similarly, IHC results demonstrated higher expression of NILCO components and targets in type II EmCa from Chinese women (Figure 3(a)).  Figure (3b) shows that Notch1, Notch4, DLL4, JAG1, and IL-1R tI had the greatest expression in type II EmCa compared to type I EmCa (*p* < 0.01).

Additionally, NILCO components showed different cellular localization patterns among type I and type II EmCa from African-American and Chinese patients (Table 2). Overall, type II EmCa from African-American patients showed higher percentage of cells with membrane-cytoplasmic staining than type I EmCa for Notch1, Notch2, JAG1, DLL4, OB-R, and IL-1R tI. Approximately 60% of cells were positive for cytoplasmic staining for Notch1 in type II (intensity: 2-to-3+) versus 30% (intensity: 1+) in type I patients (*p* = 0.001). Similarly, 74% of cells (intensity: 2-to-3+) stained positive for Notch4 in type II patients when compared to type I patients showing 24% (intensity: 1+) (*p* = 0.001). There was a significant difference in cytoplasmic staining for OB-R between type I and type II (39%, intensity: 1+ and 70%, intensity: 2+, resp.). Nuclear staining for Notch4 and Survivin had significant differences between the tumor types with type II patients showing a higher percent of positive cells (Notch4: 20%, (intensity: 2-to-3+) versus 5%, (intensity: 1+); Survivin: 75% (intensity: 3+) versus 51% (intensity:1+) (*p* = 0.0001).

Interestingly, the staining pattern for EmCa from Chinese women indicates that 93% of cells had positive cytoplasmic staining for Notch1 (intensity: 2-to-3+) in type II tumors compared to only 74% positive cytoplasmic in type I tumors (intensity: 1+; *p* = 0.0001). Majority of cells (88%) showed cytoplasmic staining for DLL4 (intensity: 2-to-3+) in type II patients (*p* = 0.0454). Additionally, OB-R and IL-1R tI were detected higher in the cytoplasms from type II (OB-R: 64%, intensity: 2+ and IL-1R tI: 90%, intensity: 2+) versus type I EmCa (OB-R: 30%, intensity: 1+; IL1-R tI 75%, intensity: 1+; *p* = 0.0001).

### 3.3. Western Blot (WB) Analyses

**Figure 4:**
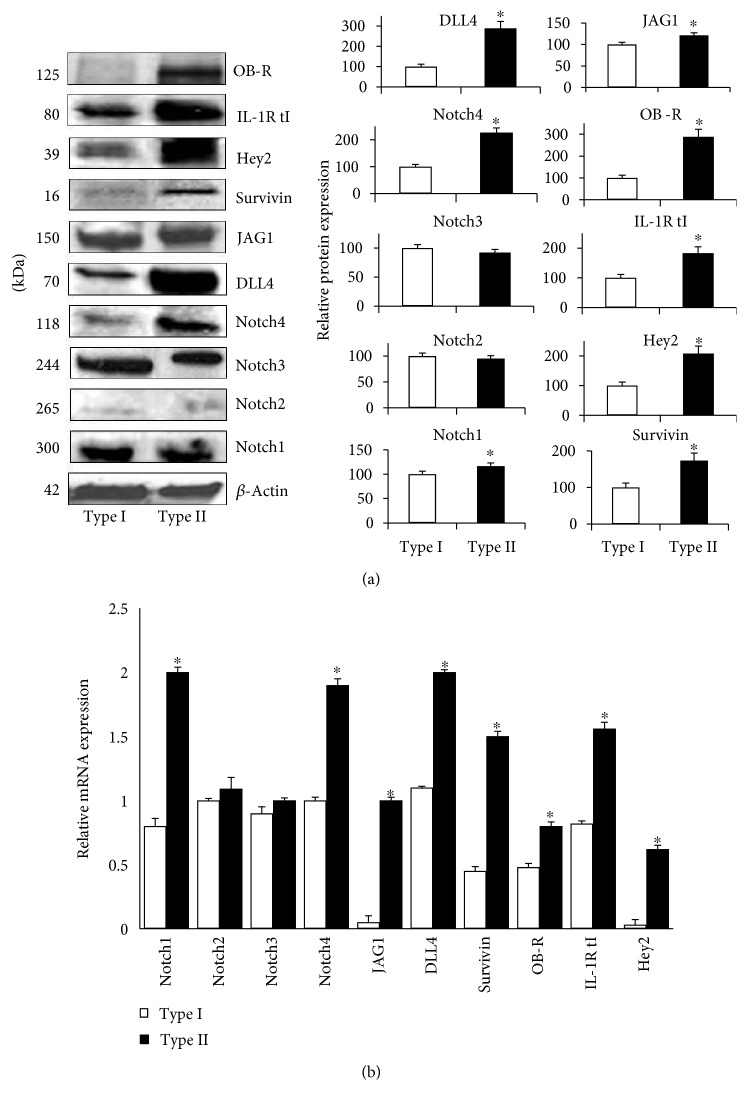
Protein and mRNA expression of Notch receptors, ligands, and molecular targets in type I and type II EmCa from African-American women. (a) Western blot representative data of NILCO components in African-American women. Type I (*n* = 8); type II (*n* = 6). (b) mRNA expression levels of NILCO and targets in African-American women by real-time PCR. mRNA levels were normalized to GAPDH. ^∗^*p* < 0.05.

Next, WB analysis was used to further determine whether NILCO and targets were expressed differently in type I and type II EmCa from African-American patients (Figure 4(a)). Type II EmCa from African-American patients showed significantly higher expression levels of Notch receptors (Notch1 and Notch4), ligands (JAG1 and DLL4) and targets (Survivin, Hey2, IL-1R tI, and OB-R). Similar results were observed for activated NICDs of Notch1–3 (results not shown). Additionally, Notch4, DLL4, OB-R, and Hey2 showed the greatest differences in expression between EmCa types, with type II patients showing at least a 2-fold difference (*p* < 0.01). Interestingly, OB-R protein levels detected by WB were virtually absent in type I EmCa that confirmed IHC results (OB-R HSCORE = 1.1 that indicates very weak detection of the antigen).

### 3.4. mRNA Levels

mRNA levels of NILCO in EmCa from African-American patients (Figure 4(b)) exhibit a similar pattern of expression as observed in WB analyses. Figure (4b) shows higher mRNA expression of Notch1, Notch4, DLL4, OB-R, and IL-1R tI in type II EmCa (*p* < 0.01). Notch1 and JAG1 mRNA showed 2-fold higher expression in type II EmCa. Interestingly, DLL4 and Hey2 mRNA both had a 20-fold change difference in expression between the two tumor types. In addition, OB-R mRNA was 3-fold higher in type II EmCa.

### 3.5. ELISA Results

**Figure 5:**
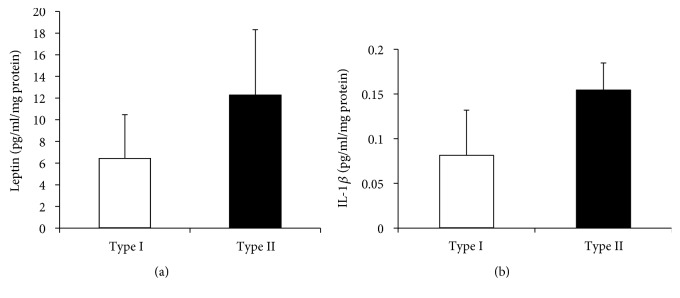
Leptin and IL-1*β* levels in EmCa lysates. (a) Leptin levels in EmCa. (b) IL-1*β* levels in EmCa. Type I EmCa (*n* = 8) and type II EmCa (*n* = 6) lysates were analyzed by ELISA (R&D systems).

No significant differences were found for leptin levels in tumor lysates from African-American patients. However, type II EmCa tends to have higher concentrations of the oncogenic adipokine compared to type I EmCa lysates (Figure 5(a)). Similar results were found for IL-1*β* levels (Figure 5(b)).

### 3.6. Effects of NILCO Inhibition on Leptin-Induced EmCa Cancer Cell Invasion

**Figure 6:**
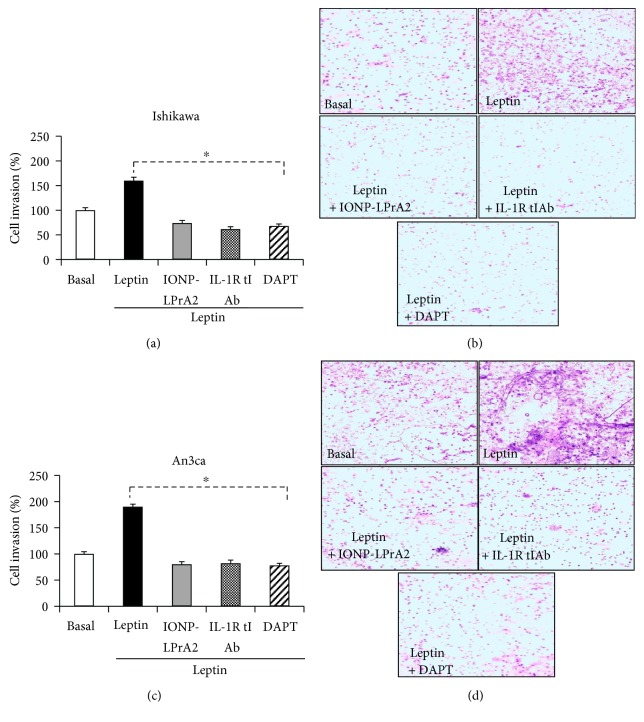
Leptin-induced EmCa cell invasion was abrogated by inhibition of Notch and IL-1 signaling. (a) Quantitative assessment of Ishikawa (derived from type I EmCa) cell invasion. (b) Representative pictures of the effects of leptin and NILCO inhibitors on Ishikawa cell invasion. (c) Quantitative assessment of An3ca (derived from type II EmCa) cell invasion. (d) Representative pictures of the effects of leptin and NILCO inhibitors on An3ca cell invasion. Results from cell migration (Boyden chamber cell invasion assay; Corning BioCoat Matrigel invasion chamber) were obtained after 24 h and normalized to basal conditions. Invading cells were detected by hematoxylin staining (see Section 2). Data (mean ± standard error) representative results derived from a minimum of 3 independent experiments. ^∗^*p* < 0.05.

To examine whether a functional NILCO signaling pathway occurs in EmCa, we determined whether leptin-induced cell invasion is affected by the inhibition of Notch and IL-1 signaling in vitro (DAPT; a *γ*-secretase inhibitor and anti-IL-1Rt I antibody). Additionally, the specificity of leptin's effects on cell invasion was assessed using a leptin receptor inhibitor (IONP-LPrA2).  Figure 6 shows that leptin significantly induced invasion in Ishikawa (Figure 6(a)) and An3ca cells (Figure 6(b)). Remarkably, leptin's effects were prominent in the more aggressive An3CA EmCa cell line that was derived from a type II EmCa [28]. Notably, the effects of leptin were abrogated by the inhibition of Notch and IL-1 signaling.

## 4. Discussion

Current data illustrate that NILCO molecules are expressed differently in EmCa types. Notably, NILCO proteins and mRNAs were expressed higher in type II EmCa, which strongly suggests that NILCO expression correlates with the development of the more aggressive form of the disease. Higher expression of NILCO was consistently found in type II EmCa biopsies from African-American and Chinese patients. Type II EmCa showed the highest expression of Notch1 and Notch4. Although, a small number of African-American EmCa patients were evaluated, obese African-American patients (*n* = 17) showed the highest levels of NILCO expression when compared to lean EmCa patients (*n* = 12). Unfortunately, lack of data on BMI and body weight made it impossible to elaborate on the potential impact of obesity on NILCO expression in EmCa from Chinese patients. These patient cohorts were different than African-American cohort, showing an unequal proportion of type I and type II EmCa that makes it difficult to establish an adequate comparison. However, it was observed that NILCO mean expression was higher in African-American women than in Chinese women suffering from EmCa.

A strong correlation between obesity and EmCa incidence has been consistently reported. EmCa is more than three times as common in obese women when compared to normal healthy weight women [31]. After menopause, progesterone synthesis is drastically reduced. However, in postmenopausal women, the adipose tissue can increase estrogen levels through the aromatization of the androgens. Obesity is characterized by high levels of leptin and estrogen, which increases cancer growth. Leptin was positively associated with EmCa [32]. Then, increased EmCa incidence and progression are related to elevated levels of estrogens (unopposed estrogen stimulus), insulin growth factor-1, adipokines (leptin, resistin), and cytokines [1]. Estrogen may influence leptin synthesis in a tissue- and cell type-specific fashion [33]. Moreover, OB-R and estrogen receptor (ER) are coexpressed in cancer indicating a possible interaction between leptin and estrogen systems to promote carcinogenesis. It was recently reported that OB-R was downregulated during the progression of EmCa, which correlated with ER and progesterone receptor expression patterns [34]. Thus, leptin/OB-R and estrogen/ER signaling pathways may work together to accelerate the progression of type I EmCa. However, in contrast to a previous report [34], we found that type II EmCa expresses higher levels of OB-R. Remarkably, type II EmCa shares genomic features with basal-like breast cancer [8], which shows low levels or absence of ER, progesterone, and EGFR2 (Her2) receptors. Basal-like breast carcinomas (triple negative) are very aggressive and have poor prognosis. Aberrant Notch signaling has been strongly associated with triple negative breast cancer. Moreover, Notch signaling is a target linked to poor prognosis in breast cancer [14]. Leptin upregulates the IL-1 system in endometrial cancer cells [25] and the Notch pathway in breast cancer [14]. A crosstalk between Notch, IL-1, and leptin signaling (NILCO) has been associated with breast cancer growth [14, 15, 23]. Type II EmCa are independent of ER signaling, very aggressive, have poor prognosis, and no targeted therapies. Therefore, NILCO may be related to type II EmCa progression [1].

It has been suggested that Notch signaling could be important for endometrial proliferation [24]. Previous studies have shown that Notch2 signaling could play a protective role in breast cancer [35]. However, the role of Notch signaling is poorly understood in EmCa [36]. Moreover, previous reports showed inconsistent endometrial expression of Notch. Increased protein expression of Notch1, Notch3, DLL4, and JAG1 was previously reported in EmCa [37]. Though, a different study reported low Notch4 expression in EmCa [38]. However, there no previous reports comparing the expression of Notch and other NILCO molecules between type I and type II EmCa. Present data show that NILCO molecules (Notch1, Notch2, Notch3, Notch4, DLL4, and JAG1) and targets (Survivin, Hey2, IL-1R tI, and OB-R) were expressed in EmCa regardless of ethnicity and cancer type.

Notch1 and Notch4 were expressed significantly higher in type II EmCa from both African-American and Chinese patients when compared to type I EmCa. Additionally, Notch ligands DLL4 and JAG1 were also expressed higher in all type II EmCa patients. Furthermore, OB-R and IL-1R tI were expressed higher in type II EmCa from African-American patients. qPCR analyses corroborated IHC and WB data on type II EmCa. Although, no significant differences were found, leptin and IL-1*β* levels within tumor tissues were higher in type II EmCa than in type I EmCa. Moreover, leptin-induced invasion of EmCa cells in vitro was remarkably higher in the more aggressive cell line An3Ca (type II EmCA) [28], which was abrogated via inhibition of Notch and IL-1 signaling. Taken together, these data suggest that functional NILCO signaling might be involved in type II EmCa progression.

Notch ligands are mainly found membrane bound as the signaling cascade is initiated upon binding of ligands to transmembrane Notch receptors expressed in adjacent cells [17]. Interestingly, NILCO molecules showed differential localization patterns in type II versus type I EmCa. Several NILCO molecules (Notch1, Notch2, JAG1, DLL4, OB-R, and IL-1R tI) were found to be expressed higher in the cellular membrane and cytoplasmatic compartment in type II EmCa from African-American patients. Additionally, type II EmCa biopsies showed higher expression of nuclear Notch4 and Survivin in these patients. Meanwhile, type II EmCa from Chinese patients showed higher cytoplasmatic levels of Notch4, DLL4, JAG1, and IL-1R tI. To the best of our knowledge, there are no reports on the cellular localization patterns of NILCO molecules in EmCa. However, the implications of the current findings warrant further investigations.

Biomarkers are promising tools for early detection and monitoring the progression of EmCa [39]. Among these biomarkers, Ki67 proliferation marker is increased in high-grade tumors. Also, abnormal activation of *β*-catenin has been observed in type I EmCa [40, 41]. Serum and plasma biomarkers (i.e., CA125) have been explored in EmCa development and progression, but no specific tumor markers have been investigated for obesity-related type II EmCa [42]. Current data may suggest a potential for NILCO expression as a novel biomarker for type II EmCa. Higher NILCO expression in EmCa might indicate the more aggressive disease and a potential new target for treatment.

Data from the present study should be interpreted considering some limitations: (1) We did not establish selection criteria for the patient cohort to collect EmCa samples from African-American patients. Rather, the EmCa samples were obtained from current patient population attending to the Gynecological Medical services at Grady Hospital; (2) We were only able to collect a small number of EmCa samples from African-American women but not from Caucasian women since the patients attending Grady Hospital are predominantly African-American; and (3) The cohort of Chinese patients suffering from EmCa was obtained from a company that provided commercial tissue microarrays and does not fully specify on the clinicopathological characteristics of patients, that is, BMI and body weight. Despite these study limitations, we could present the first preliminary evaluation of NILCO expression in type I and type II EmCa.

## 5. Conclusion

Although, there are some controversial opinions on how to clearly define type I and II EmCa, it is generally recognized that type II EmCa comprises a phenotype of malignancy characterized by the lack of hormonal response, high aggressiveness, and poor prognosis. EmCa incidence and progression are conclusively affected by obesity. Currently, there are no consistent data reported on the expression of Notch in type I and type II EmCa. NILCO components are expressed higher in breast cancer and other cancer types [41]. Present data strongly support the notion that type II EmCa expresses higher NILCO molecules, which suggests that the more aggressive and nonresponsive hormonal disease could be dependent on leptin-Notch-IL-1 signaling. Furthermore, current data indicate that obesity induces higher NILCO expression in EmCa that might be linked to leptin signaling. Additionally, inhibition of Notch and IL-1 signaling in vitro significantly reduced leptin-induced invasion of EmCa cells. Therefore, leptin could induce the more malignant phenotype via promoting the expression and crosstalk between Notch and IL-1 in EmCa. Current observations deserve further investigations including higher number of samples from different ethnic groups of lean and obese patients. These investigations might lead to the identification of NILCO as a novel target and biomarker in EmCa, particularly for type II EmCa.

## Figures and Tables

**Table 1:** Histopathological characteristics of EmCa in African American and Chinese women.

	African-American			Chinese		
	Type I (*n* = 12)	Type II (*n* = 17)	*p* value	Type I (*n* = 97)	Type II (*n* = 23)	*p* value
Age(±SD)	54.45 (±9)	67.02 (±6)	0.008	53 (±10)	57 (±13)	0.1439
BMI	29.81 (+3)	34.17 (+2)	0.034	n/a	n/a	

	*n* (%)	*n* (%)		*n* (%)	*n* (%)	

Grade I	6 (50)	0 (0)	0.001	44 (45)	2 (9)	<0.001
Grade I~II	n/a	n/a		14 (14)	0 (0)	
Grade II	6 (50)	0 (0)		2(2)	0 (0)	
Grade II~III	n/a	n/a		15 (16)	9 (39)	
Grade III	0 (0)	17 (100)		22 (23)	12 (52)	
Stage I	9 (75)	5 (29.4)	0.0051	83 (86)	12 (52)	<0.001
Stage II	0 (0)	1 (5.9)		9 (9)	3 (13)	
Stage III	3 (25)	6 (35.3)		5 (5)	5 (22)	
Stage IV	0 (0)	5 (29.4)		0 (0)	3 (13)	

Data are presented as percentage of number (*n*) of EmCa from African-American and Chinese women. Age range is presented as mean (+SD). The *p* value was calculated by two-sample *t*-test for age, grade, and stage between tumor types. BMI: body mass index.

**Table 2:** Differential cellular staining pattern of NILCO and targets in type I and type II EmCa tissues from African-American and Chinese women.

	African-American			Chinese		
	Type I (*n* = 12)	Type II (*n* = 17)	*p* value	Type I (*n* = 97)	Type II (*n* = 23)	*p* value
	% positive	% positive		% positive	% positive	
*Notch1*						
Nucleus	1	1	0.0806	4	10	0.0779
Cytoplasm	30	60	0.0001	74	93	0.0184
*Notch2*						
Nucleus	1	3	0.0001	12	11	0.2259
Cytoplasm	85	90	0.0001	79	91	0.0121
*Notch3*						
Nucleus	1	4	0.0001	5	0	0.0001
Cytoplasm	71	64	0.0001	46	56	0.0001
*Notch4*						
Nucleus	5	20	0.0001	4	12	0.0522
Cytoplasm	24	74	0.0001	68	63	0.5882
*JAG1*						
Nucleus	2	2	0.5501	1	14	0.0001
Cytoplasm	80	94	0.0001	72	63	0.3446
*DLL4*						
Nucleus	1	6	0.0001	15	39	0.0001
Cytoplasm	81	84	0.0061	72	88	0.0454
*Survivin*						
Nucleus	51	75	0.0001	42	58	0.0450
Cytoplasm	30	19	0.0001	82	84	0.8104
*OB-R*						
Nucleus	0	2	0.0001	0	0	n/a
Cytoplasm	39	70	0.0001	30	64	0.0001
*IL-1R tI*						
Nucleus	1	3	0.0001	1	1	0.6359
Cytoplasm	55	63	0.0001	75	90	0.0001
*Hey2*						
Nucleus	1	1	0.6224	0	0	n/a
Cytoplasm	90	90	0.7111	100	100	0.6841

Nuclear and cytoplasmic staining for NILCO and targets are expressed as % of positive immunoreactivity in type I and type II EmCa from African-American and Chinese women. Notch1–4 (Notch receptor type 1 through 4); JAG1: Jagged1; DLL4: Delta-like ligand 4 (Notch ligands); Survivin, Hey2 (hairy and enhancer of split-related protein 2), IL-1R t1 (interleukin-1 receptor type 1), and OB-R (leptin receptor). The *p* value was calculated by two-sample *t*-test.
